# Insights from the comparisons of SARS-CoV and COVID-19 outbreaks: The evidence-based experience of epidemic prevention in China

**DOI:** 10.1097/MD.0000000000024650

**Published:** 2021-02-12

**Authors:** Wen-Yi Liu, Yen-Ching Chuang, Ting-Jun Liu, Ching-Wen Chien, Tao-Hsin Tung

**Affiliations:** a Department of Health Policy Management, Bloomberg School of Public Health, Johns Hopkins University, Baltimore, MD, USA; b Shanghai Bluecross Medical Science Institute, Shanghai; c Institute for Hospital Management, Tsing Hua University, Shenzhen Campus; d Tai Kang Institute of Healthcare Management, Beijing; e Department of Medical Research and Education, Cheng-Hsin General Hospital, Taipei, Taiwan; f Maoming People's Hospital, Maoming, Guangdong, China.

**Keywords:** coronavirus disease 2019 outbreak, severe acute respiratory syndrome coronavirus

## Abstract

Coronavirus disease 2019 (COVID-19) is one of infectious diseases caused by severe acute respiratory syndrome coronavirus 2 (SARS-CoV-2). At the beginning of 2020, a sudden outbreak of novel pneumonia, originated from Wuhan, China, swiftly evolves to a worldwide pandemic, alike the severe acute respiratory syndrome (SARS) in 2003. However, Chinese-style innovation in response to the outbreak of COVID-19 helped China to reach a faster and more effective success in the containment of this epidemic. This review summarizes insights from the comparisons of severe acute respiratory syndrome coronavirus (SARS-CoV) and COVID-19 outbreaks on the basis of preventive strategies in China for this coronavirus pandemic.

## 1 Introduction

Coronavirus disease 2019 (COVID-19) was first identified in December 2019 in Wuhan, the capital of China's Hubei province, and has since spread to the whole China in terms of large-scale transportation, resulting in the ongoing 2019–2020 coronavirus pandemic. At this globalism time, the virus is likely to overwhelm most countries.^[1]^ As of April 6^th^, COVID-19 has already attacked 183 countries or regions in the world, with the epicenter swiftly transferring from China to Italy and America.^[2]^ The areas influenced by COVID-19 are 6 times higher than severe acute respiratory syndrome (SARS).^[3]^ Promisingly, pandemic will grow to be a great threat to human health like malarial, tuberculosis and acquired immune deficiency syndrome (AIDS). However, it still remained unclear how, when, where, and why a pandemic would happen, though the trajectories of them were seemingly the same according to the World Health Organization (WHO).^[4]^ Hence, we must learn lessons from each fight and establish an early warning mechanisms to give a sensitive, specific, and flexible response. Early detection by integrated screening regimens followed by appropriate clinical intervention may offer a practical means for the prevention of condition-associated infectious damage.

## 2 Clinical perspective to COVID-19

Although COVID-19 is inferior to SARS in terms of symptoms and mortality, it requires more attention due to the following reasons:

### 2.1 More contagious

COVID-19 patients may be most infectious in the days before they began showing symptoms. A person with COVID-19 may be contagious 48–72 hours before starting to experience symptoms. Patients without symptoms may be more likely to spread the illness, because they are unlikely to be isolating and may not adopt behaviors designed to prevent spread. A protein mutation grants COVID-19 a higher infectivity than severe acute respiratory syndrome coronavirus (SARS-Cov), especially during incubation.^[5,6]^ For the “Diamond Princess” a cruise ship, a tourist without clinical symptoms eventually led to 621 other cases in less than a month.^[7]^ The virus that causes COVID-19 could be much more contagious than believed previously. Versatile transmission channels, among which droplets and close contact are verified, while aerosol and fecal-oral routes are still undetermined, contribute to the fast spread of this novel coronavirus pneumonia.^[8]^

### 2.2 Become a pandemic

Pandemics are states of an infectious disease that significantly increase in populations around the world with infections occur more or less simultaneously. The pandemic of COVID-19 has caused significant social and economic disruption in the world, including the largest global recession since the Great Depression. Within a month, 34 provinces, municipalities, and special administrative regions in Mainland China discovered the detected cases.^[9]^ On March 11th 2020, the WHO announced the outbreak of COVID-19 to be a worldwide Pandemic after an initial declaration as a Public Health Emergency of International Concern.^[10,11]^ As of April 6th 2020, COVID-19 has spread to 183 countries and regions, involving a total of 1,289,380 confirmed cases.^[2]^ Compared with SARS, which infected 8,422 people in 25 countries or regions across 5 continents,^[12]^ the world now is suffering from a much hard times.

### 2.3 Longer period

COVID-19 may have a longer incubation period in comparison with the 7-month SARS outbreak, suggests probability analysis of Wuhan cases.^[13]^ By applying the renewal theory in disease probability to reduce recall bias in initial case reports, scientists have come up with a new estimate approach for the incubation period of COVID-19. From the first concentrated pneumonia cases reported on December 29^th^, 2019, in the city of Wuhan in China's Hubei province,^[14]^ it took 3 months to clear new cases on the mainland.^[15]^ North America and Europe have now become the new centers of the outbreak. Based on the current trajectory, the WHO predicts that COVID-19 may last until 2021.^[16]^

### 2.4 Broader consequences

From the economic viewpoint, China's gross domestic production was 4% of the global total in 2003, when battling with SARS. However, now it accounts for 17% as the second biggest economy in the world.^[17]^ Due to the outbreak of COVID-19, the possibility of hitting a 30-year low in national growth has risen. People may experience decreased income from several social distancing ways. Most of people could not work at home, especially those in public facing roles in service industries, a sub-group already faces precarious employment and low income threaten. Others may be affected by workplace closures, caused by government mandate, an infected co-worker, or loss of business.^[18]^ In the UK, 3.5 million additional people are expected to need universal credit (which includes unemployment payments) as a result of the pandemic of COVID-19.^[18]^ In addition, as the world's largest manufacturer and importer of crude oil, China's situation has triggered a chain reaction, even a global recession.^[19,20]^ US stocks are currently suffering from their biggest fall since 1987, and the “circuit breaker” is affecting 11 countries and regions.^[21]^

### 2.5 Clinical guidance for COVID-19

Recommended preventive measures for COVID-19 include hand washing, covering one's mouth when coughing, keep social distances, wear a face mask in public areas, disinfecting surfaces, ventilating and air-filtering, and monitoring and self-isolation for people who suspect infected. The Chinese National Health Commission has published 7th version “Chinese Clinical Guidance for COVID-19 Pneumonia Diagnosis and Treatment” to indicate deeper understanding of the clinical manifestations and pathological features of this infectious disease and ”the accumulation of experience in diagnosis and treatment." This guidance reviews the epidemiology, etiology, clinical features, clinical classifications, and management of COVID-19 for adults and children population. In addition, for the severe and critical types of COVID-19, it shows information how to differentiate COVID-19 infections and pneumonia from upper respiratory tract infections or other known viral pneumonias. Clinical guidance also provides for identifying cases and filing reports, as well as appropriate treatment. The document not only outlines recommendations for general treatment, but also applies treatment of severe and critically severe cases, and options for discharge criteria and precautions.

## 3 Literature review

It is estimated that 80%∼85%^[22]^ of patients who contracted COVID-19 did not become severely ill, with some even being asymptomatic. However, these asymptomatic carriers still have the ability to infect others,^[5]^ making COVID-19 quite different from SARS. Thus, establishing an early warning system, developing effective testing kits, taking strong measures to isolate the source of infection, and employing a hierarchical governance model are of great importance to containing the outbreak.

### 3.1 The management and health policy of COVID-19 in China

China performed better this time around due to a quicker response and more data transparency. It was not until April 20th, 2003, six months after the first case of SARS was reported in Foshan, Guangdong, on November 16th, 2002, that SARS information was made public.^[23]^ Misinformation spreading via mobile phones caused widespread panic and mass buying of vinegar, radix isatidis, antibiotics, and food.^[24]^ In contrast, when the first unknown pneumonia case occurred in Wuhan on December 8^th^, 2019, measures, such as collecting virus samples, conducting an epidemiological investigation, and releasing therapeutic regimens, were taken within 45 days to enact a quick response to the emergency. Eight rumor-mongers were dealt with according to the law. An online data center almost a year after the first case appeared confirmed cases in China.^[4]^

The Chinese National Medical Products Administration (NMPA) help bring more efficient and sensitive testing kit to the market faster. Although polymerase chain reaction (PCR) protocols were proposed since the initial completion of the SARS genome sequenced by a Canadian group on April 13, 2003,^[25]^ efficient and reliable PCR diagnostic assay, orienting clinical application, was not available until October, almost a year after the first case appeared.^[26]^ In striking contrast, attributed to NMPA's emergency procedure, it took COVID-19 testing kits based on the reverse transcription polymerase chain reaction (RT-PCR) technology only half a month to be applied in clinical quarantine. With the availability of the Real-Time Fluorescent RT-PCR test, a coronavirus detection could be completed in a three-hour turnaround time.^[27]^

China also made good use based on limited medical resources with utilizing the mobile emergency hospital. In February 2020, COVID-19 entered its most severe period in Wuhan.^[28]^ Medical resources, especially wards, were in serious shortage, even though Huoshenshan, Leishenshan, and Xiaogangshan (Huanggang and Beijing). Special hospitals could provide a sum of 5,100 beds^[29,30]^ successively came into use. Referring to the successful rescue work that was done during the Wenchuan and Yushu earthquakes, the Chinese government proposed building large-scale mobile emergency hospitals to facilitate, treat, and monitor patients with minor symptoms.^[31,32]^

The use of the mobile emergency hospitals began in the 1960 s. In the beginning, it was a mobile medical platform with integrated medical and technical support functions that could be rapidly deployed in the field. It was first designed by the U.S. military to treat the wounded and sick in Vietnam. When the 21^st^ century came around, mobile emergency hospitals gradually began to take part in allocating medical resources during disaster relief and non-war military operations as well. In China, the government also made good use of limited medical resources, leaving top-level devices available to treat more severe cases by utilizing mobile emergency hospitals.^[33,34]^ 16 mobile emergency hospitals were set up in Wuhan within two weeks. By March 10th, when all mobile emergency hospitals were taken down, they had provided 13,000 beds and treated more than 12,000 patients, equal to a quarter of all COVID-19 patients in Wuhan.^[30]^ In contrast, during the SARS outbreak in 2003, the Xiaotangshan reconstructed hospital treated a total of 680 cases, or just 1/7th of the confirmed cases in China.^[35]^

### 3.2 The strategy of lockdown in China

China put 16 cities into lockdown for the first time since 1949 due to prevent infections of COVID-19.^[36]^ Compared with SARS first appearing in November of 2003, COVID-19 first appeared in December of 2019, close to the annual Spring Festival, an important traditional Chinese holiday characterized by family reunion.^[37]^ Based on the data from 2019, 2.98 billion trips were made during the 40-day Spring Festival travel rush, with an average of 70 million trips a day concentrated around New Year's Eve.^[38]^

Before Wuhan was isolated, a research model estimated the possibility of COVID-19 being transported from Wuhan to 369 other cities in China. In 130 cities (95% CI 89–190), the expected COVID-19 risk is > 50% and > 99% in the four largest metropolitan areas.^[39]^ During this time, all forms of public transportation, such as long-distance bus routes, metros, express railways, and aviation were uncompromisingly forbidden.^[40]^ This was the first time China simultaneously put 16 cities into lockdown since its founding. Second-generation epidemic centers, including Wenzhou and Taizhou, all took strict actions to restrict movement as well. By February 20th, statistical data from airsavvi showed that China had canceled a total of 10,126 domestic flights and 2,628 international flights.^[41]^

**Figure 1:**
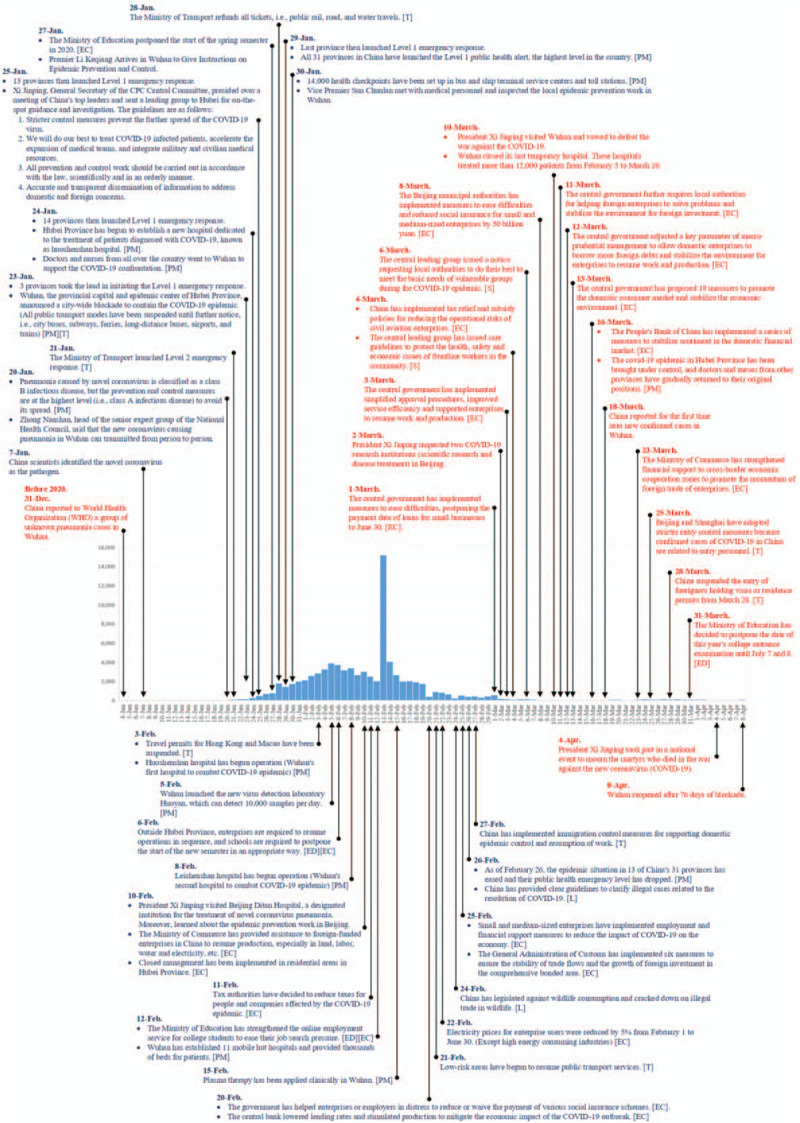
The epidemic curve of confirmed COVID-19 cases^[42]^ and its corresponding control measures in China^[43,44]^.

Under the extensive, stringent containment, a hierarchical governance model was able to guarantee the launch and implementation of all policies. The Chinese National Health Commission set up a special command group to coordinate all COVID-19 affairs. This command group arranged China's overall response framework during COVID-19. Local governments took action regarding the corresponding policies and situations, and district governments receive detailed arrangements from local governments. Personnel were then dispatched to solve problems, such as recording the population of people in Wuhan and its surrounding areas. It is worth to notice that during the disease outbreak, community leaders and active neighborhood members took initiative to help district governments isolate patients, food delivery, check temperatures, and report information. University students working as volunteer translators also helped bridge the gap among foreigners. The epidemic curve of confirmed COVID-19 cases^[42]^ and its corresponding control measures in China,^[43,44]^ are showed in Figure 1.

## 4 The comparisons of SARS-CoV and COVID-19 outbreaks

### 4.1 Epidemiological spreading

SARS, a global pandemic infectious disease, first occurred in Guangdong Province, China. It lasted nine months before, totally eliminated from November 2002 to July 2003. The outbreak spread to the whole China from Guangdong, except for seven provinces, including Tibet, Xinjiang, and Qinghai. Moreover, the epidemic gradually overwhelmed a total of 25 countries or regions, mainly affected southeast Asia, Australia, Europe, and North America.^[12]^

**Figure 2:**
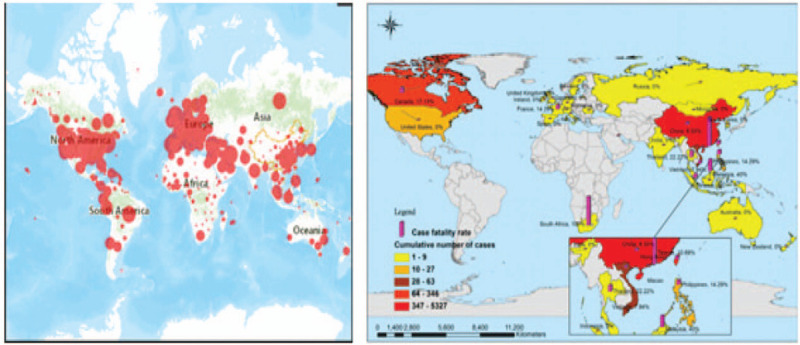
Global mapping of coronavirus disease COVID-19^[2]^ and SARS-Cov^[3]^.

**Figure 3:**
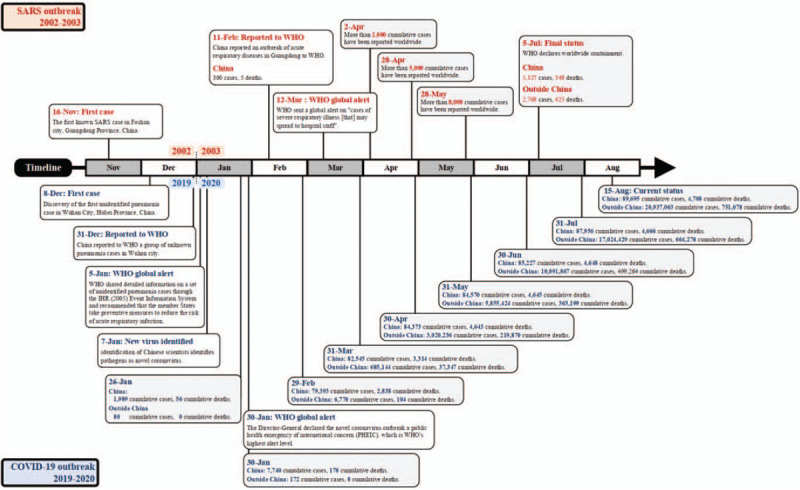
The seasonal distribution of cases of SARS-CoV v.s COVID-19^[42,46,47]^.

Seventeen years later, another pandemic, COVID-19, sweep all over the world. No more than a month did it spread to 34 provinces, cities, or districts in China, since the first case of pneumonia of unknown aetiology identified in Wuhan, one of the most populous cities in central China. The outbreak outside of China happened later, overwhelming over 6 times countries or regions than that during SARS. Japan, South Korea, Singapore, France, the United States, Australia were badly hurt, notably, Egypt, on the African continent, declared unaffected during SARS could also be an outbreak ensue. Furthermore, contrast with SARS, COVID-19 was featured by a surge of the epicenter successively or spontaneously.^[2,45]^Figure 2 indicates the Global mapping of coronavirus disease COVID-19^[2]^ and SARS-Cov.^[3]^Figure 3 showes the seasonal distribution of cases of SARS-CoV v.s COVID-19.^[42,46,47]^

### 4.2 Chinese-style innovation during COVID-19 deserved a global concern

Under rigorous containment and quarantine efforts, China became the first country to dampen the epidemic. Chinese approach has been recognized and supported by some contries when confronted with this severe public health emergency, which spread faster, covered wider, and more difficult to prevent and control in comparison to SARS.^[48]^

#### 4.2.1 A rational utilization of mobile emergency hospital could relieve the pressure of medical resources shortage

To date, never did a pandemic, such as the European plague, tuberculosis, cholera, African Ebola, and Spanish influenza in the middle ages become under control via drug therapies, but vanishing before the specific drug approached to clinical use, home and abroad.

The use of mobile emergency hospital rapidly curbs viral spread, while the receivable collection policy also ensured that most should receive active treatment of patients to get the best quality of medical resources allocation. Mobile emergency hospital got birth as early as in the Vietnam War, with a view to helping lightly wounded soldiers return to the battlefield as soon as possible, while it works as a temporary medical unit, transferring medical equipment in the rear hospital to the rescue site to avoid missing the best time for treatment to a great extent when disasters happen, nowadays.^[49]^ Mobile emergency hospitals in Wuhan did some improvements to the original concept through appropriately endowing large public facilities such as sports hall, exhibition hall, and school with medical adaptations. They provided large capacity for the treatment of mildly patients and centralized management and rehabilitation,^[50]^ which brought Wuhan, the birthplace and epicenter of COVID-19, under control in just three months.^[51]^

Although mobile emergency hospital plays an important role in public health emergencies, it is not suitable for long-term large-scale use regardless of cost.

#### 4.2.2 The essential of accident insurance

Insurance for medical staff is a major innovation during the novel coronavirus.

During SARS period, medical workers did not receive insurance equivalents to the potential risk of their jobs or adequate financial compensations, especially when they were infected or even died on duty. Xiaoling Wei, a nurse at Guangzhou women's and infant hospital, died with her husband from SARS. However, their 8-year-old son could only get 450 RMB (Renminbi, Chinese dollars) a month from the hospital.^[52]^ In COVID-19 outbreak, the Chinese government attaches great importance to protecting the legitimate rights and interests of medical workers and has introduced a series of policies, including increasing temporary work subsidies and increasing health and epidemic prevention subsidies.^[53]^ More than 20 insurance companies in China donated 100,000–1,000,000 RMB of superimposed insurance to medical workers at the frontline.^[54]^ Take doctor Wenliang Li as an example, after his death in the line of duty, 24 insurance companies prepared exclusive insurance for him, amounting up to 11.6 million RMB, of which 1.3 million RMB was immediately sent to his family.^[55]^

#### 4.2.3 The function of military management and volunteers

Military management and volunteers involved in retaining a highly-efficient hierarchical government. Xiaotangshan-style military management plays an indispensable role in halting further viral spread during both SARS and COVID-19. In response to novel pneumonic higher infectiousness compared to SARS, each province and city in China had to recruit much more volunteers than they did in 2003, especially medical or linguistic students on campus to solve the shortage of manpower. As of 30^th^ March 2020, more than 30 thousand volunteers have engaged in prevention and quarantine, working a total of over 18.2 million hours, nearly 60 hours averaged.^[56]^

In the absence of vaccines and antiviral drugs, only strict implementation of traditional public health measures can curb the prevalence of this respiratory diseases. Community-wide containment is an intervention measure applied to the whole community, city or region. Its implementation requires close partnership and cooperation with law enforcement departments at the local and state levels.^[57]^ As a result, the presence of volunteers promptly ensured the implementation of specific isolation measures in communities, schools, and fundamental organizations

### 4.3 Weakness and future improvement

Although it has been 17 years since the end of SARS, China still did not take it all in its stride when COVID-19 came in this spring. We need to do improvements in the following aspects:

#### 4.3.1 Early warning for infectious diseases and public health emergencies is insufficient

It is worthy to note that the measures China took to treat and settle suspected or detected novel pneumonia patients were of reference value for the whole world. However, obvious flaws exist in early warning. Established after SARS, the Chinese surveillance system for infectious diseases and public health emergencies failed to send the early warning report directly to the Chinese Center for Disease Control. Consequently, we lost the best opportunities to dampen the outbreak from the very beginning. It was not until 20^th^ January, nearly 2 months after the first case announced, did the State Department introduce COVID-19 to the direct online reporting system.^[58]^

#### 4.3.2 The overall level of protective equipment reservation and rescue aids allocation should be enhanced

Considering the cost of operation, the majority of hospitals were prone to keeping no more than one-month protective supplies at regular time, notably, some of them even ignore the preparedness. Due to a safe seventeen-year time without any large-scale public health, incidents happening in China, the government, hospitals and Red Cross Organizations gradually lack the consciousness that the army marches into its stomach so that they did not pay enough attention to the stock of medical protective equipment, which led to a serious shortage of medical supplies in this outbreak.^[59]^

#### 4.3.3 The logistics support mechanism needs to be further improved

Owing to local logistics units’ bureaucratic matching and distribution procedures, shortages of protective equipment such as masks and protective clothing could be frequent to see in the designated hospitals, though numerous national rescue aids arrived in Wuhan immediately when the epidemic got worsen.^[60]^ In addition, the logistic work for the personal safety of medical workers has been neglected, and medical incidents caused an adverse impact on the diagnosis and treatment of the epidemic.^[61]^

### 4.4 Further improvement

Infectious diseases such as influenza, in terms of WHO, will become one of the greatest threats to human health in the future. It is difficult for us to predict when, where, or even from whom they will arrive.^[62]^

#### 4.4.1 Introduce a sensitive, specific, and flexible early warning mechanism into China

**Figure 4:**
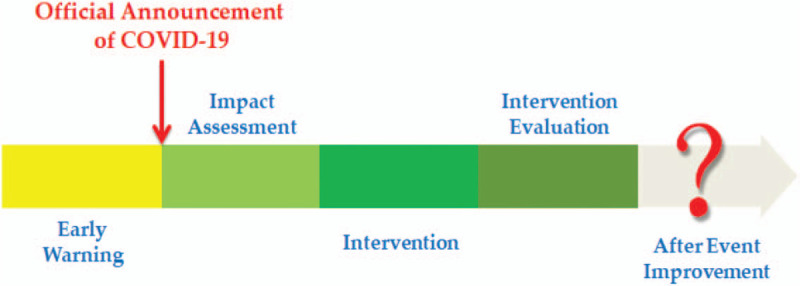
The management process of COVID-19.

Early warning mechanism is developed with the view to solving problems probably occurring in public health outbreaks emergencies, like inaccurate information, slow response, and inadequate preparation. Figure 4 shows the management process of COVID-19. This infectious disease matched the Wilson criteria for screening due to it is an important health problem; the disease natural history should be understood; a recognisable latent or early symptomatic stage; a test is easy to perform and interpret, acceptable, accurate, reliable, sensitive and specific; an accepted treatment recognised for the disease; treatment is more effective if started early; a policy on who should be treated; diagnosis and treatment are cost-effective; and case-finding should be a continuous process.

#### 4.4.2 Optimize the matching and distribution procedure

In sight of local logistics units’ lagging and inefficient performance during the outbreak this spring, they should exalt staff's professionalism and have a good command of the actual needs in areas under administration, to a great extent, making the allocation rational. A multi-channel donating model should also be conducted in emergency. Local logistics units not only must be Hospitals, but also should know the approaches to protective and therapeutic equipment, guaranteeing the quantity reserved in accordance with national criterions.

#### 4.4.3 Reduce the possibility of getting infected on the way to China for overseas

The concept of wearing masks means different to diverse cultures, which escalated the cross contract possibility in sealed public transportation space, especially airlines. To date, the American government utilized a containerized bio-containment system to help people evacuate safely from disaster areas to locations where appropriate medical treatment can be received, which should be learnt by universal world.^[63]^

## 5 Conclusion

It is worth noting that with the development of COVID-19, China has successfully become the first country to control the epidemic under rigorous containment and quarantine efforts. The epidemic curve of confirmed cases and the results of corresponding control measures (Fig. 1) also showed that China has provided a feasible overall management mode for major infectious diseases, including public health, medical treatment, economy, transportation, education, and other policies. This management model can serve as a reference basis for countries to face this major infectious disease and control models.

However, according to the WHO Coronavirus Disease (COVID-19) dashboard, by 4:37 p.m. CET on December 3, 2020, 63,965,092 confirmed COVID-19 cases, including 1,488,120 deaths, had accumulated worldwide. Among them, the number of confirmed cases in most regions has not shown an obvious downward trend, which also highlights that the world still has a long way to go before COVID-19 can be effectively controlled.

At the same time, we should learn from the lesson that in the fight against this major infectious disease, there are obvious management defects in the early warning mechanism, protective equipment reserve, auxiliary equipment allocation and logistic support mechanism. In the future, it is important to establish a well-functioning early warning mechanism and management so as to make a more sensitive and flexible response to the next pandemic. The government should have systematic thinking and put forward a set of management measures from the perspective of social operation and from different aspects such as medical treatment, transportation, education and economy. The hospital further optimized the configuration and configuration procedures of protective articles, and paid attention to the regular inspection and storage of protective articles.

## Author contributions

Wen-Yi Liu, Ching-Wen Chien, and Tao-Hsin Tung conducted the study and drafted the manuscript. Yen-Ching Chuang and Ting-Jun Liu participated in the design of the study. Wen-Yi Liu, Tao-Hsin Tung, and Ching-Wen Chien conceived the study, and participated in its design and coordination. All of the authors read and approved the final manuscript.

**Conceptualization:** Wen-Yi Liu.

**Methodology:** Ting-Jun Liu.

**Supervision:** Ching-Wen Chien.

**Writing – original draft:** Wen-Yi Liu.

**Writing – review & editing:** Yen-Ching Chuang, Ching-Wen Chien, Tao-Hsin Tung.

## Abbreviations

Abbreviations: AIDS = acquired immune deficiency syndrome, COVID-19 = coronavirus disease 2019, NMPA = National Medical Products Administration, PCR = polymerase chain reaction, RT-PCR = reverse transcription polymerase chain reaction, SARS = severe acute respiratory syndrome, SARS-CoV = severe acute respiratory syndrome coronavirus, SARS-CoV-2 = severe acute respiratory syndrome coronavirus 2, WHO = World Health Organization.
